# Updates of Pathogenesis, Diagnostic and Therapeutic Perspectives for Ovarian Clear Cell Carcinoma

**DOI:** 10.7150/jca.53395

**Published:** 2021-02-22

**Authors:** Chenchen Zhu, Zhihao Xu, Tianjiao Zhang, Lili Qian, Weihua Xiao, Haiming Wei, Tengchuan Jin, Ying Zhou

**Affiliations:** 1 Department of Obstetrics and Gynecology, Anhui Provincial Hospital, Anhui Medical University, Hefei, 230001, China.; 2 Department of Obstetrics and Gynecology, The First Affiliated Hospital of USTC, Division of Life Sciences and Medicine, University of Science and Technology of China, Hefei, Anhui, 230001, China.; 3 Division of Molecular Medicine, Hefei National Laboratory for Physical Sciences at Microscale, The CAS Key Laboratory of Innate Immunity and Chronic Disease, School of Life Sciences, University of Science and Technology of China, Hefei, China.; 4 Institute of Immunology, University of Science and Technology of China, Hefei, China.

**Keywords:** Ovarian clear cell carcinoma, epithelial ovarian carcinoma, endometriosis, targeted treatment

## Abstract

Ovarian clear cell carcinoma (OCCC) is a special pathological type of epithelial ovarian carcinoma (EOC) and has a high prevalence in Asia without specific molecular subtype classification. Endometriosis is a recognized precancerous lesion that carries 3-fold increased risk of OCCC. Ovarian endometrioid carcinoma, which also originates from endometriosis, shares several features with OCCC, including platinum resistance and younger age at diagnosis. Patients with OCCC have about a 2.5 to 4 times greater risk of having a venous thromboembolism (VTE) compared with other EOC, and OCCC tends to metastasize through lymphatic vesicular and peritoneal spread as opposed to hematogenous metastasis. There is only mild elevation of the conventional biomarker CA125. Staging surgery or optimal cytoreduction combined with chemotherapy is a common therapeutic strategy for OCCC. However, platinum resistance commonly portends a poor prognosis, so novel treatments are urgently needed. Targeted therapy and immunotherapy are currently being studied, including PARP, EZH2, and ATR inhibitors combined with the synthetic lethality of ARID1A-dificiency, and MAPK/PI3K/HER2, VEGF/bFGF/PDGF, HNF1β, and PD-1/PD-L1 inhibitors. Advanced stage, suboptimal cytoreduction, platinum resistance, lymph node metastasis, and VTE are major prognostic predictors for OCCC. We focus on update pathogenesis, diagnostic methods and therapeutic approaches to provide future directions for clinical diagnosis and treatment of OCCC.

## Introduction

Epithelial ovarian carcinoma (EOC) is the most lethal gynecologic cancer and ranks as the seventh leading cause of cancer-related women's deaths worldwide 1, 2. In China, ovarian cancer is the third most common gynecologic cancer, ranking behind cervical and uterine cancers. The incidence is higher in rural residents compared with women in urban populations 3. Ovarian clear cell carcinoma (OCCC) is a specific pathological type of EOC with unique clinical and molecular features. Patients usually present with a large, unilateral pelvic mass, and occasionally have thromboembolic vascular complications or hypercalcemia 4, 5. The 5-year overall survival (OS) and progression-free survival (PFS) rates are 80%~89% and 56%~88% for International Federation of Gynecology and Obstetrics (FIGO) stages I and II and decrease to 52% and 25% for stages III and IV, respectively 6, 7. OCCC patients at late stage tended to have poorer prognosis than those with other histological subtypes of EOC, especially in advanced EOC stages with 1.65-fold higher hazard rate for death 8. Meanwhile, though serum CA125 levels are frequently mildly or moderately elevated, CA 125 is a poor diagnostic of OCCC 9. Thus, to improve the ability to diagnose OCCC, novel serological biomarkers are necessary to be identified. Because OCCC displays chemoresistance to platinum, the efficacy of platinum-based chemotherapy is only 20% to 50% for OCCC, so more precise therapy is urgently needed 6, 10, 11. In this review, we summarize current research progress and treatment strategies for OCCC with the aim of aiding the clinical diagnosis and treatment of OCCC.

## Epidemiology

EOC is the most lethal gynecologic cancer, resulting in estimated 239,000 new cases and 152,000 related deaths worldwide every year. In China, it is the tenth most common cancer among women and although the incidence is relatively low (4.1/100,000), because China has such a large population, it is estimated that there are still 52,100 and 22,500 cases of women diagnosed with or dead of EOC in 2015, respectively 1-3. Generally, up to 90% of ovarian carcinomas are classified as EOCs and they are divided into the following subtypes based on histopathology: high-grade serous carcinomas (HGSC), accounting for 70% of all EOCs; low-grade serous carcinomas (LGSC), accounting for <5%; endometrioid, accounting for 10%; clear cell, accounting for 10%, and mucinous, accounting for 3%, making up more than 95% of all EOCs 12.

The prevalence of OCCC differs by region. It accounts for 5%~10% of EOC in North America and 12% in other western countries, but it has a higher prevalence in East Asia, accounting for 25%~30% and 10.3%~11.6% of EOCs in Japan and Korea, respectively 13-15. Due to the highest rate prevalence in Japan, a study revealed that there may be an association between OCCC and ZNF217 amplification among Japanese patients, which may promote neoplastic transformation by promoting cell survival during telomeric crisis 16.Morbidity also differs by race, which is higher in Asians (11.1%) and lower in black, white, and other populations (3.1%, 4.8%, and 5.5%, respectively) 17.

## Risk factors

Compared with HGSC, patients with OCCC tend to be diagnosed at a younger age (56 years vs 75 years) and a lower FIGO stage 18, 19. The association of obesity and the risk of EOC has been reported, but it is weak in OCCC (odds ratio [OR] = 1.06 per 5 kg/m^2^) 2. In addition, endometriosis is significantly related to the pathogenesis of both ovarian clear cell and endometrioid carcinomas: an increased risk of OCCC (OR = 3.05) and ovarian endometrioid carcinoma (OR = 2.04) among women with endometriosis has been identified by Pearce 20.

Several reproductive and hormonal risk factors are also linked to OCCC, including early menarche, late menopause, low use of oral contraceptives, and low pregnancy rate. This may be because these women have had more ovulations and led to more cellular divisions to repair epithelium after each ovulation, potentially resulting in a greater number of spontaneous mutations and malignant transformations 21-23.

The intrauterine device (IUD) is the most common method of contraception in China, used by about 50% of all women of reproductive age. The widespread use of IUD and its strong contraceptive effect may have benefit to reduce the incidence of EOC in China. The specific detailed mechanisms between IUD and EOC remain unclear, differences in the type of IUD as well as the usage time of IUD may also make a difference associated to the risk of EOC. 24, 25. Pregnancy leads to anovulation and suppression of the secretion of pituitary gonadotropins, which likely has a protective effect on women, particularly for OCCC and ovarian endometrioid carcinomas, with 50% to 70% of decreased risk, compared with 20% reduction for serous carcinoma 2. Hysterectomy and tubal ligation also have been identified associated with a decreased risk of OCCC, ranging from 30%-40%, and the proposed biological mechanisms include limiting the retrograde menstruation and the elevation of inflammatory agents 2, 26, 27.

OCCC shows little association with family history, and BRCA1/ BRCA2 germline mutations are uncommon in OCCC (2.1%) 28. Conversely, ARID1A (which target AT-rich interactive domain 1A) somatic mutations and PIK3CA (phosphatidylinositol-4,5-bisphosphate 3-kinase catalytic subunit alpha) occur frequently in OCCC 29, 30. The other genetic alterations and possible molecular targets in OCCC are presented in **Table 1.** In addition, ARID1A and PIK3CA mutations have been found to occur early in tumorigenesis of OCCC 31, Loss of ARID1A expression was usually coincident with PI3K-AKT pathway activation and/or ZNF217 amplification which contributed to the development of OCCC 32. OCCC is frequently positive for HNF1β (>95%), and it is negative for estrogen receptors (ERs) and Wilms Tumor 1 (WT1) in more than 95% of cases 12, 33, 34. Recently, Yang et al. detected mutations of MUC4 (28.6%), MAGEE1 (19%), and ARID3A (16.7%), which have not been previously reported, and MAGEE1 mutation predicts a poorer outcome 35. Compared with other histological subtypes of EOC, OCCC has a distinct methylation profile, including synchronous gain of promoter methylation for multiple genes in the ER alpha pathway and loss of promoter methylation for numerous genes in the HNF1 pathway 36. However, further investigation will be required to more precisely outline the functions of these two pathways in this disease.

## Molecular Classification of OCCC

It is known that the four molecular subtypes were identified in HGSC, immunoreactive, differentiated, proliferative and mesenchymal, on the basis of gene expression in the clusters. Winterhoff et al also have validated the transcriptional subtypes on OCCC, they suggested that the OCCC group at advanced stage could use this same transcriptional profiling of HGSC while the OCCC group at early stage may have distinct transcriptional signatures 37. Similarly, the PROMISE diagnostic algorithm is a reliable surrogate of the molecular group in endometrial carcinoma and ovarian endometrioid carcinoma, including p53, mismatch repair (MMR) protein immunohistochemistry, and DNA polymerase ε (POLE) exonuclease domain mutation. The role of these markers in OCCC have also been explored, and found that most of OCCC patients have normal p53 expression, only a few of OCCC patients with abnormal p53 expression had adverse features and poor prognosis. However, low frequency of MMR abnormalities and no pathogenic POLE mutations were found in this research. Thus, the role of PROMISE algorithm remains to be elucidated 38. Since somatic mutations of ARID1A loss have been frequently identified in OCCC, classified OCCC based on ARID1A expression status also helped to distinguish distinct subtype of OCCC. ARID1A-positive tumors were more likely to be histologically of high grades, ERβ-positive, HNF1β-negative and E-cadherin-negative than ARID1A-negative tumors, but without difference of age, parity, tumor stage and cancer-specific survival 39. However, BAF250a encoded by ARID1A is a member of the SWI/SNF complex, aggressive behaviors and poor prognosis were observed in the OCCC losing one or multiple SWI/SNF complex subunits 40. Two OCCC gene expression subtypes were identified through gene expression profiles: epithelial-like (EpiCC), which is associated with early-stage disease, with a relatively higher rate of gene mutations in the SWI/SNF complex; and mesenchymal-like (MesCC), associated with late-stage and poorer PFS but higher enrichment of immune-related pathway activity as well as preferential drug response to bevacizumab, which could be helpful for prognostic and therapy 41. Uehara et al. performed single nucleotide polymorphism analysis, and they suggested that expression profiles might be useful for sub-classification of OCCC. Type A was a cluster with broad range and low frequency of copy number alterations (CNAs), type B was a cluster with broad range and low to high frequency of CNAs, and type C was a cluster with focal range and high frequency of CNAs. Endometriosis and early stage were more commonly observed in cluster A than in clusters B/C, but with lower overall response rate to platinum-taxane chemotherapy 42. In conclusion, there is no clear and specific molecular typing method suitable for OCCC, it is still required to further investigate novel and reliable molecular subtype classification of OCCC.

## Pathogenesis of OCCC

### Atypical endometriosis is a precancerous lesion of OCCC

It is common in reproductive-age women, occurring in 5% to 10% of women at 25 to 35 years old. The association of endometriosis and ovarian cancer has been widely reported 43, 44. Between 18% and 43% of women with OCCC have a history of endometriosis 19, 45-48, and several studies have demonstrated that this benign disease is a precursor lesion of OCCC and endometrioid carcinoma 21.

**Figure 1:**
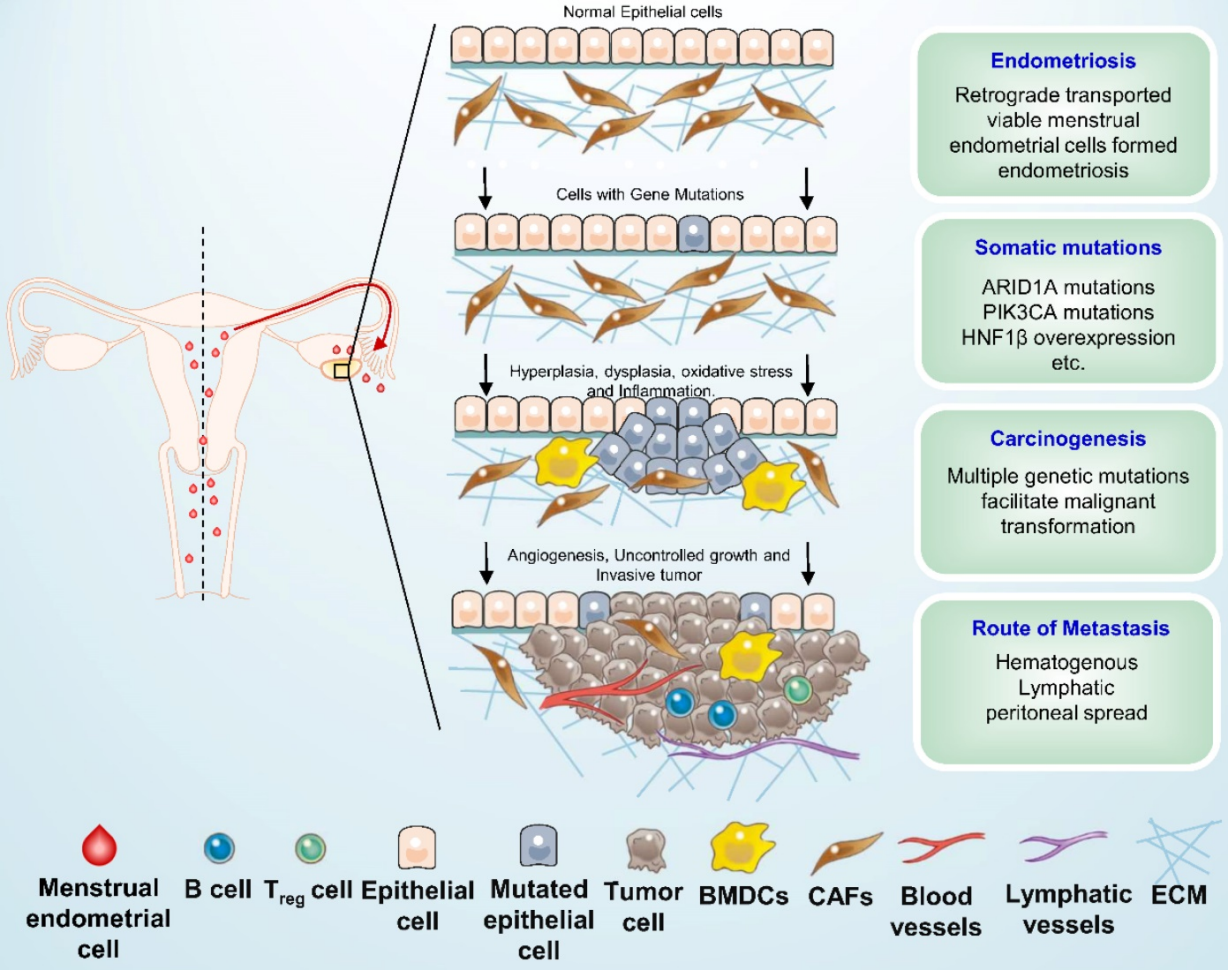
** Schematic overview of ovarian clear cell carcinoma development.** Shed menstrual endometrium leaves the cavity and retrograde along the fallopian tube to the ovary and into the pelvic or abdominal cavity (red arrow), it may then form endometriosis under multiple factors. Several genetic alterations, such as ARID1A, PIK3CA mutations and HNF1β overexpression, as well as some microenvironmental change, were suspected to be associated with early carcinogenic events of ovarian clear cell carcinoma. BMDCs: Bone marrow-derived dendritic cells; CAFs: Cancer-associated fibroblasts; ECM: Extracellular matrix.

Several hypotheses have been advanced to account for the association between endometriosis and ovarian carcinoma. The most well-known of these is the implantation theory, which posits that the viable menstrual endometrial cells were deposited in the pelvic cavity via retrograde menstruation and became the origin of ectopic endometrial tissue. These shed menstrual endometrial cells still capable to attach to the peritoneum, invade, proliferate, and differentiate 49. The ovary is probably favored seeding sites for endometriosis cells especially in the ovulation sites 21. Women with endometriosis have been reported to have a two- and three-fold increased risk of OCCC and endometrioid carcinoma, respectively 20. Inflammation of coelomic epithelial cell-derivatives in the female reproductive tract is a major contributor to malignant transformation in endometriosis-associated OCCC 50. The microenvironment in endometriosis contains elevated local IL-6 production as well as high oxidative stress which is caused by ions release from disrupted heme 51, and eventually contributes to genomic damage. Some somatic mutations have been detected in paired eutopic and ectopic endometrium, and ectopic tissue has a higher mutation burden 52. Endometriotic lesions commonly carry multiple somatic mutations; atypical endometriosis and co-existing tumors share nearly all of the somatic mutations, such as high expression of HNF1β and driver mutations in ARID1A and PIK3CA, and it is thought that those above mutations occurred early in the malignant transformation of the OCCC 21. In addition, ARID1A and PIK3CA mutations were found to cooperate to promote tumor growth through sustained IL-6 overproduction, and IL-6 was identified as a physiological target of ARID1A tumor suppressor activity 50. The schematic diagram of the pathogenesis of OCCC is presented in **Figure 1.** Moreover, endometriosis was found to be associated with amplification of epidermal growth factor receptor (EGFR) gene and the activation of EGFR plays a critical role in cell proliferation, apoptosis, angiogenesis, and metastasis 16, thus it is necessary to identify whether the amplification of EGFR can trigger the progression from endometriosis to carcinoma. The risk of tumorigenesis in endometriosis is about 1% among premenopausal women and 1% to 2.5% among postmenopausal women 45, 53. The risk for 20-year-old women is considered to be 1.00, therefore the risks for women in their 40s and 50s are 3.60 and 10.7, respectively, which indicates that the tumorigenesis of endometrial cysts occurred around menopause 54. However, Anglesio et al. reported that deep infiltrating endometriosis (DIE) has a low risk of malignant transformation because the somatic driver mutations tend to be confined to the epithelial compartment 55. Recently, six functional gene clusters in pathogenesis network of OCCC were uncovered by integrated analysis of transcriptomes, including ribosomal protein, eukaryotic translation initiation factors, lactate, prostaglandin, proteasome, and insulin-like growth factor 56. And Su et al. suggested that complement-activation-alternative-pathway may be the crucial dysfunctional immunological pathway in duality for carcinogenesis at all OCCC stages 57. Nevertheless, further investigation needs to be proceeded about the essential pathogenesis of endometriosis and OCCC.

### Comparison of Ovarian Endometrioid Carcinoma and OCCC

OCCC and ovarian endometrioid carcinoma are 2 pathologic subtypes of EOC, which both likely arise from ovarian or pelvic endometriosis and share some similarities 21, 58. **Table 2** compares the characteristics of these 2 cancers. Both ovarian endometrioid carcinoma and OCCC are identified as Type I tumors which progress in a stepwise manner, whereas HGSC is identified as a Type II tumor with an aggressive phenotype and without specific indications 2. Under the premise of endometriosis, the risk of OCCC and endometrioid carcinoma increases by 2~3 times 20, but there is no association between survival and endometriosis in either carcinoma 58, 59. Endometrioid carcinoma, accounting for 5% to 10% of all EOCs, is frequently diagnosed when it is at a low grade and early stage with a better prognosis than other pathologic EOC subtypes at the early stage, similar to OCCC 18, 19, 60. However, both endometrioid and clear cell carcinomas have low rates of platinum sensitivity, which contributes to their poor prognoses when they present at a late stage or as a recurrence 48, 58.

In spite of their similarities, ovarian endometrioid carcinomas and OCCC should be identified as different diseases with unique clinical and molecular characteristics 48, 59, 61. It is suggested that low- and intermediate-grade endometrioid carcinoma is clinically and biologically different from the high-grade stage 62, which is apparently similar to HGSC, sharing the common feature of p53 mutations and homologous recombination repair deficiencies 63. However, OCCC is usually not recommended to grade based on morphological features, without prognostic significance 64. The course of chemotherapy for these 2 cancers is also different, as reflected in the postoperative treatment guidelines issued by the National Comprehensive Cancer Network (NCCN), 2020, version 1 65. For ovarian endometrioid carcinoma, observation is optional for patients with stage IA through IC (grade 1) and stage IA through IB (grade 2). Adjuvant chemotherapy or hormone therapy is optional for stages II through IV (grade 1) disease and chemotherapy is considered for stage IC (grade 1) and substages IA through IC (grades 2 and 3) diseases. For patients with grade 2 or 3 endometrioid carcinoma above stage II, the treatment principle is the same as for HGSC. Moreover, OCCC patients showed a higher hazard ratio for death than ovarian endometrioid carcinoma for all stages 8. As for OCCC, chemotherapy or observation is alternative for substage IA, and adjuvant chemotherapy is recommended for all the other stages in NCCN guideline. In terms of molecular characteristics, a higher frequency of ARID1A mutation has been detected in OCCC (46% to 57%) than in endometrioid carcinoma (30%) 47. Whereas phosphatase and tensin homolog (PTEN), Kirsten rat sarcoma (KRAS), and p53 and β-catenin gene mutations are more often in ovarian endometrioid carcinoma 59.

### The mechanism hypothesis of the difference In OCCC and Ovarian Endometrioid Carcinoma

To date, it is unclear how endometrioid carcinoma and OCCC, 2 histologically and clinically different tumors, can arise from the same tissue, i.e., the endometrial epithelium of ovarian endometriosis. More and more evidence indicates that OCCC and endometrioid carcinoma possibly originate from different types of endometriosis cells 66. Kajihara et al. hypothesized that OCCC might originate from already existed endometriosis resulted by retrograde menstruation, and ovarian Mullerian metaplasia might induce tumorigenesis of endometriosis-associated endometroid carcinoma. Another hypothesis was put forward by Cochrane et al. that OCCC is derived from a ciliated cell lineage, whereas endometrioid carcinoma is derived from a secretory cell lineage 67. It has been suggested that the tumorigenesis of EOC might via 2 modes: a hormone-independent pathway for OCCC and a hormone-dependent pathway for endometrioid carcinoma 68 - on the basis of the low ER expression in OCCC and higher in endometrioid carcinoma. As a result of repeated hemorrhages in endometriosis, the interactions between iron-mediated oxidative stress and the low ER expression are thought to be associated with the tumorigenesis of OCCC 48. In addition, Davis et al. found that 41.4% of patients with ovarian endometrioid carcinoma associated with endometriosis had synchronous endometrioid cancer, far more than the 3.8% that is associated with OCCC 69. Therefore, they hypothesized that high levels of estrogen leading to the proliferation of endometriosis are implicating in the carcinogenesis of endometriosis.

## Clinical characteristics of patients with OCCC

Patients with OCCC usually examined with a huge unilateral pelvic mass confined to the ovary, accompanied by abdominal pain and swelling symptoms 70 with a mild-to-moderate elevation of serum CA125 6, 70. Particularly, they are likely to develop hypercalcemia 4, 5, resulted from the elevated expression of the parathyroid-hormone-related protein (PTHRP) and the activation of stanniocalcin-1 signaling mediated by IL-6 71.

In Son's study, nearly 75% of the cases of OCCC were confirmed to have an association with endometriosis, and endometriosis tend to be diagnosis in women at the age of late 30s and 40s, afterwards, malignant transformation would be completed with a median of 4 years 53. Endometriosis usually occurs as a unilateral ovarian cyst, OCCC tumors tend to grow intracystically and confined to the ovary until spreading for a long time. They usually have symptoms such as pelvic mass, dysmenorrhea, and dyspareunia, who could be better followed up with frequent hospital visits, while women with other EOC usually have no symptom until they reached advanced stages. These factors mean that patients with OCCC tend be diagnosed with earlier stage in younger age, without ascites or positive peritoneal cytology 48, 72. Women with no symptom who are diagnosed with OCCC when periodic physical examination are also tend to be at an earlier stage with a smaller tumor size 53. In ultrasound examinations and computed tomography (CT) of OCCC often reveal a huge, well-defined, unilateral mass with solid components and cyst fluid or necrotic portions 73, 74. Recently, Stukan et al. found that preoperative lung and intercostal upper abdomen ultrasonography performed in patients with EOC can add valuable information for supradiaphragmatic and subdiaphragmatic metastases 75.

Pathologically the OCCC lesions are usually present as huge masses consist of solid tissue that protrudes into the cyst cavity and commonly displays a combination of papillary, tubulocystic, and solid microscopic patterns. The tumor invades the ovarian interstitium, causing desmoplasia, stromal destruction, hyalinization, desmoplasia and confluence of the epithelial elements. 73. The presence of clear cells alone could not directly confirm OCCC because clear cytoplasm could also be found in cells of ovarian endometrioid carcinoma and HGSC. OCCC characteristically contains clear or hobnail cells with eccentric, rounded, and bulbous nuclei, multiple complex papillae, densely hyaline basement membrane material, and hyaline bodies. Compared with other types of EOC, the frequency of mitoses is lower (usually < 5 /10 HPFs) 12.

## Venous thromboembolism

**Figure 2:**
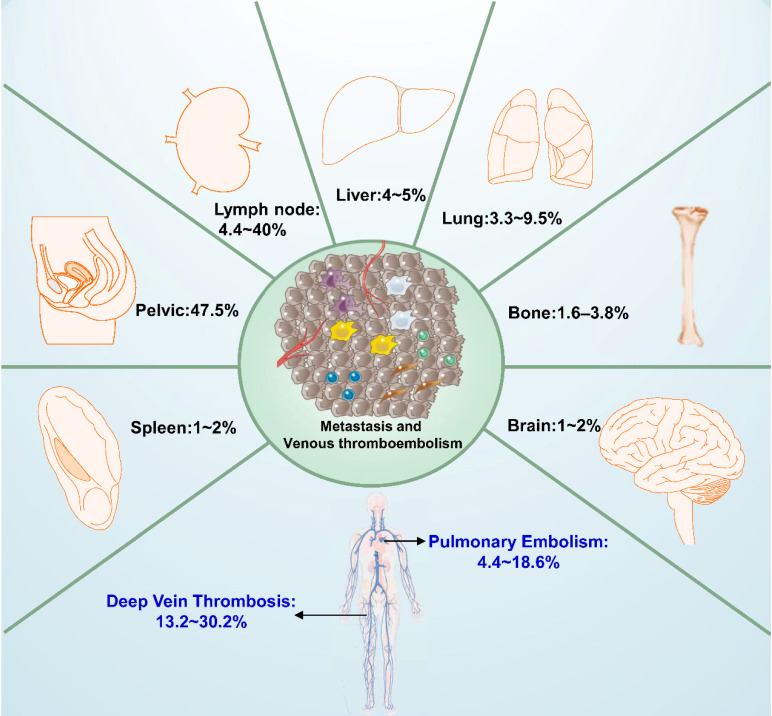
** Distributions of metastatic lesions and the commonly complication (venous thromboembolism, VTE) of ovarian clear cell carcinoma.** Patients with ovarian clear cell carcinoma especially at advanced stages have high recurrence rates, hematogenous, lymphatic and peritoneal spread are general routes to metastasis. The most frequent sites of metastasis are lymph node and pelvic cavity, rarely in brain, bone and spleen. VTE, consist of deep vein thrombosis (DVT) and pulmonary embolism (PE), is the common complication in epithelial ovarian carcinomas especially in ovarian clear cell carcinoma.

Venous thromboembolism (VTE), consist of deep vein thromboses (DVTs) and pulmonary embolisms (PEs), is common in EOCs because of its intrinsic malignancy and time-consuming operations 76, 77. The incidence of VTE in EOC patients has been reported to be between 1% and 26%, and the incidences of DVT and PE are 11~18% and 1~2.6%, respectively 78, 79 (**Figure 2**). Patients with OCCC have a higher risk of VTE (15% to 42%), PE (4.4% and 18.6%), and DVT (13.2% and 30.2%), about 2.5 to 4 times higher than is seen in other subtypes 77, 80-82. VTE is more commonly seen in advanced-stage OCCC (21.9%) compared with early-stage (8.2%) disease, and PE is more common at advanced stage disease (10-fold). VTEs in patients with advanced OCCC tend to occur in the proximal veins, such as the postcaval, iliac, femoral, and popliteal veins 77. The elevated IL-6 expression and frequent alteration of tissue factor pathway inhibitor-2 might increase the risk of VTE in OCCC 71, 83. And HNF1β was found associated with glycogen metabolism, including glucose-6-phophatase, and strikingly the blood clotting cascade, including fibrinogen, prothrombin and factor XIII. Positive HNF1β was significantly linked to a 3.0-fold increased risk of clinically-significant venous thrombosis among gynecologic carcinomas with cytoplasmic clearing 84.

In terms of timing, most VTE events are found at the initial examination, before the primary surgery (36.4%), and developed when the disease reccurs or progresses (33.3%). They can also occur following the primary adjuvant chemotherapy period (18.2%) and postoperatively (12.1%) 77, 85. Based on our review and the studies of other researchers, there are some appropriate measurable biomarkers for the increased risk of VTE in ovarian cancer, such as elevated platelet counts, d-dimer levels, white blood cell counts, and CA125 levels, and decreased hemoglobin and albumin levels in the preoperatively; as well as elevated d-dimer levels and decreased albumin levels postoperatively 86-88. Aggressive operations and chemotherapy are also potential risk factors for VTE 89. For example, lymphadenectomy can damage the vascular epithelium, promoting the formation of VTE. Therefore, extended thromboprophylaxis should be suggested for patients receiving chemotherapy or having lymphadenectomy 89, 90. Because VTEs can occur despite of appropriate prophylaxis, a more aggressive postoperative anticoagulation regimen and prolonged post-discharge VTE prophylaxis should be considered for patients with OCCC 81.

## Tumor markers

### Serum cancer antigen 125 (CA125)

The conventional tumor marker CA125 has long been used in the diagnosis of HGSC. It is elevated in 75.6% of serous carcinoma cases but in only 57.6% of OCCC cases 91. Thus, CA125 is a poor marker for OCCC, with only a mildly elevated baseline value and a frequent incidence of false-negative results 9, 92. However, CA-125 levels can be used for predicting advanced stage disease, suboptimal debulking and platinum-resistance with cut-off values of ≥ 46.5 U/mL, ≥11.45 U/mL, and ≥66.4 U/mL 93. Increased CA125 levels after the end of chemotherapy is significantly associated with shorter PFS and OS, so it also can be used as a valid indicator of the prognosis and efficacy of chemotherapy in patients with OCCC 94. Because there is currently no appropriate biomarker for OCCC, novel diagnostic markers are urgently required to improve early diagnosis and therapeutic stratification of the disease to provide more favorable prognoses and survivability.

### Systemic Inflammatory Response (SIR) markers

Inflammation is a sign of tumor, and tumor-related inflammatory microenvironments promote tumor growth and metastasis. Previous studies have confirmed that chronic inflammation have an effect on tumorigenesis and response to therapy 95, 96, and further affect the prognosis. Platelets also can produce some factors related to tumor growth, invasion and angiogenesis, contributing to protect tumor cells from natural killer cell-mediated lysis and tumor cells metastasis 93. Several SIR biomarkers are found in peripheral blood. For example, the neutrophil-to-lymphocyte ratio (NLR), lymphocyte-to-monocyte ratio (LMR), and platelet-to-lymphocyte ratio (PLR) have been reported as potential biomarkers in different cancers 97.

In patients with OCCC, high NLRs are associated with advanced-stage disease, intraperitoneal metastasis, more ascites, elevated CA-125 levels, platinum resistance, and poor prognosis 9, 93, 96, 98. Japanese researchers reported that most patients with early stage OCCC showed complete response to initial treatment that decreased NLR levels, reflecting the improvement in tumor inflammation. In patients who developed a recurrence, NLR was found to be elevated to levels as high as preoperative levels 9. Therefore, postoperative NLR may predict tumor inflammation in recurrent disease, but it may be affected by certain factors, such as the site of recurrence and the type of previous treatment. Conversely, low LMR is reportedly associated with advanced-stage disease, lymph node (LN) metastases, ascites, and low platinum sensitivity, as well as prognosis, suggesting that low LMR is due to decreased levels of peripheral lymphocytes, which weakens immune surveillance and the response to chemotherapy 96. PLR levels are not associated with the clinical characteristics of OCCC, but high PLR levels tend to be related to poor OS without significance, and PLR < 205.4 was an independent factor for the reduced risk of non-complete response 93, 96, 98. The optimized NLR, LMR, and PLR cut-off values are 2.3 to 3.3, 4.2, and 124 to 165, respectively 9, 96.

### Novel OCCC biomarker candidates

OCCC exhibits increased activity in several signaling pathways that may drive cancer: cell cycle regulation, survival, anti-apoptosis, chemoresistance, metabolism, coagulation, and angiogenesis 7. Based on the genomic alteration characteristics, it is possible to find some potential sources of diagnostic and prognostic biomarkers for OCCC, including hepatocyte nuclear factor 1β (HNF1β), expressed in almost all cases of OCCC without specific correlation with FIGO stage and is now used as a diagnostic marker to predict ovarian histological subtypes 33, 99. In immunohistochemistry (IHC), OCCC tends to be positive for CK7 and negative for CK20, hormone receptors ER and PR, WT1, and p53 12, 34, 100. In addition, negative α-fetoprotein and CD10 can used to make differential diagnoses, excluding yolk cell tumors and renal cell carcinoma 5. Left-right determination factor (LEFTY), a novel member of the transforming growth factor-β superfamily, may be an excellent OCCC-specific molecular marker with a significantly higher expression in OCCC compared with other subtype of EOC 101. MiR-509-3-5p, miR-509-5p, miR‑483‑5p and miR‑449a were significantly overexpressed whereas miR-510 and miR-129‑3p were significantly downregulated in OCCC compared with HGSC, and miR-182-5p was most overexpressed in OCCC compared with normal ovarian epithelium 102, 103. In addition, several serum biomarker candidate proteins of OCCC were identified: associated with cell cycle regulation [hepatitis A virus cellular receptor 1 (HAVCR1) and tumor protein D52 (TPD52)], growth factor signaling [insulin-like growth factor binding protein 1 (IGFBP1); KiSS-1 metastasis-suppressor; erb-b2 receptor tyrosine kinase 2 (ERBB2); and fibroblast growth factor receptor 2 (FGFR2)], anti-apoptosis and survival pathways [sialidase 3 (NEU3)], metabolism [γ-glutamyltransferase 1 (GGT1)], chemoresistance [napsin A aspartic peptidase (NAPSA), glutathione peroxidase 3 (GPX3); and aldehyde dehydrogenase 1 family, member A1 (ALDH1A1)], coagulation [coagulation factor III (F3); and tissue factor pathway inhibitor 2 (TFPI2)], signaling [lectin, galactoside-binding and soluble, 3 (LGALS3)], and adhesion and the extracellular matrix [cadherin 1, type 1, E-cadherin (epithelial); versican; and laminin, α 5(LAMA5)] 92. However, additional investigation is required to discover new elevated proteins in peripheral blood or body fluids and confirm their efficacy in the diagnosis or monitoring of OCCC.

## Metastasis

Patients with OCCC, especially those with advanced-stage disease, have high recurrence rates. Hematogenous, lymphatic, and peritoneal spread are general routes for metastasis, but the patterns of OCCC metastasis have not been specifically described 15, 104. Between 51.5% and 66.2% of recurrent stage I to III OCCC occurs in the peritoneal cavity, even though primary cytoreduction leaves no residual tumor 105. It is thought that this might be related to the spread of endometriosis into the pelvic peritoneum. Early-stage OCCC, confined in a cyst, commonly remains relatively motionless for a long time, until it pierces the cyst wall, which allows malignant cells to transported through blood vessels or the lymphatics, or to spread into the peritoneal cavity after the cyst ruptures 106. OCCC tends to metastasize more frequently through the lymphatics and spread into the peritoneal cavity rather than through the blood vessels 107. The distributions of metastatic lesions of ovarian clear cell carcinoma are presented in **Figure 2.** In Mueller's study, 4.4% to 20% of clinically apparent stage I OCCC had lymph node involved. And this rate will be higher with positive cytology or ovarian surface involvement, accounting for as much as 37.5% of metastases 108. Patients with localized relapse of OCCC tended to have a favorable prognosis (PFS=19 months, PRS=43 months). The most frequent site of recurrence was the peritoneal cavity, followed by lymph node metastases to the pelvic, para-aortic, and other lymph nodes (4.4% to 40%) and abdominal wall lesions (8.2%).

Parenchymal organ metastases were occasional in the liver (4% to 5%), lungs (3.3% to 9.5%) and spleen (1.6%), respectively 15, 105, 108, 109. The liver and lung are commonly affected organs, similar to HGSC. Splenic metastasis from EOC is uncommon, accounting for 2% to 4% of malignant spleen tumors, and it is seen more frequently in HGSC 110. Bone metastasis rarely occurs (1.6% to 3.8%) in EOC 111, and it appears to be a late stage of the disease, usually with a survival time of <4 months after radiographic diagnosis 111. However, bone metastases tend to be more common in OCCC than in HGSC, according to Jenison's study, accounting for 16% of OCCC metastases compared with no cases of bone metastases in the group of patients with HGSC 112. This is consistent with prior research, which reported that bone metastases are rare in HGSC 113, 114. Metastasis to the brain is also rare, accounting for only 1% to 2% of all EOC and most commonly seen in HGSC, with a median OS of 8.2 months 115. Brain metastasis has rarely been reported in OCCC, with only 13 cases reported as of this writing 116-119. Surgery, whole-brain radiation, stereotactic radiosurgery, gamma knife surgery, and chemotherapy can be used for metastatic brain lesions, but the blood-brain barrier could prevent effective drug delivery, which a great obstacle to chemotherapy 119.

## Treatment

Standard staging surgery or optimal cytoreduction combined with systemic chemotherapy is the usual primary therapeutic strategy for OCCC, according to the NCCN guidelines (version 1.2020). The preferred regimens of postoperative systemic therapy are paclitaxel 175/carboplatin (paclitaxel 175 mg/m^2^ iv. followed by carboplatin AUC 5-6 iv. Day1, repeated every 21 days for 3 to 6 cycles) for stage I OCCC. For stage II to Ⅳ OCCC, post-operative treatment is the same as for high-grade serous, endometrioid (grade 2, 3) carcinomas and carcinosarcoma: paclitaxel 175/carboplatin and paclitaxel/carboplatin/bevacizumab + maintenance bevacizumab (paclitaxel 175 mg/m^2^ iv. followed by carboplatin AUC 5-6 iv., and bevacizumab 7.5 mg/kg iv. Day 1, repeated every 21 days for 3 to 6 cycles, with continued bevacizumab for up to 12 additional cycles). The same regimen is also recommended for stage II to IV OCCC (ICON-7 and GOG218) 65. However, global clinical trials are needed personalized medicine for rare tumors, so that the use of bevacizumab as first-line chemotherapy for OCCC is still controversial which needs to be further validated.

### Primary Cytoreductive and Staging Surgery

Staging surgery or cytoreduction is recommended for patients with OCCC in every stage. Complete surgery with no gross residual macroscopic (R0) disease is the most important prognostic factor for OCCC. Significantly poorer prognosis has been observed even with small-volume residual disease. The prognosis was reported to be significantly better in the complete resection group compared with the groups having residual tumor diameters both greater than and less than 1 cm following the initial surgery 120. So that effort should be made to the greatest extent to remove all gross disease during an operation of OCCC. Two clinical trials, CHORUS and EORTC55971, compared the outcomes of patients with advanced EOC who had primary cytoreduction and NACT (neoadjuvant chemotherapy) + interval cytoreduction and reported noninferior survival for patients treated with NACT 121, 122. However, because the incidence of OCCC was low (1.5% to 6.0%) in both trials and because OCCC is inherently resistant to platinum, to apply these results needs more investigation specific to OCCC.

Systematic pelvic and para-aortic lymphadenectomy is important to accurately determine disease stage, provide prognostic information, and guide adjuvant therapy 15. Although these procedures have been widely used in EOC, the efficacy of lymphadenectomy is ambiguous in OCCC. It has been reported that systematic lymphadenectomy benefit to longer OS, and that the number of lymph node excision is a potential prognostic predictor for early OCCC, with PFS of the group that had ≥35 lymph nodes removed significantly better than the PFS of the group that had <35 lymph nodes removed 123, 124. However, an Italian multicenter trial illustrated that lymphadenectomy increased OS only in advanced OCCC but had no effect on survival in patients with early disease 125. Suzuki et al. also reported that there was no difference in survival for early OCCC with or without lymphadenectomy 126. It is plausible to limit the extent of lymph node dissection in selected cases, considering that the positive lymph nodes exist in only a minority of cases and that lymphadenectomy can increase the potential for complications, both intraoperatively (vascular and neurologic injury) and postoperatively (lymphedema and the formation of lymphocysts) 108. Further study should be performed to verify the efficacy of lymphadenectomy on OCCC.

### Secondary Cytoreductive Surgery

The efficacy of surgery for recurrent EOC has been debated for a long time. The DESKTOP III trial was conducted among platinum-sensitive recurrent EOC to compare secondary cytoreductive surgery plus chemotherapy with chemotherapy alone, and showed that the former resulted in an improvement PFS of 5.6 months, but this study only included a small number of patients with OCCC (<5%) 127. Kajiyama demonstrated that patients with recurrent OCCC who had re-cytoreduction had median PFS and post-recurrence survival times of 10.9 and 21.2 months, respectively, longer than the survival times of patients who only had salvage chemotherapy, but without statistic significance 105. Other studies found that patients with a solitary recurrence or metastasis to other sites that could be resected showed improved post-recurrence survival 105, 128. Thus, the choice of treatment for patients with recurrent OCCC should be based on the performance status, site of recurrence, and platinum-sensitivity.

### Fertility-Sparing Surgery (FSS)

Approximately 12% of EOC occurs in patients of reproductive age 129, so for patients desiring to remain fertile, fertility-sparing surgery that only perform unilateral salpingo-oophorectomy to preserve the unaffected ovary and the uterus, should be considered. However, the NCCN guidelines do not recommend FSS for stage IA to IC OCCC but indicate that FSS is optional for patients hoping to preserve fertility with apparent early-stage disease and/or low-risk tumors, such as early-stage invasive epithelial tumors, low malignant potential lesions, malignant germ cell tumors, mucinous tumors, or malignant sex cord-stromal tumors. However, a Gynecologic Cancer Inter Group (GCIG) consensus review indicated that FSS should not be considered for OCCC beyond stage IC 15. Previous researches have assessed the outcomes of FSS among cases of stage I OCCC. Based on the National Cancer Institute's Surveillance, Epidemiology, and End Results (SEER) data, Nasioudis considered FSS was safe for stage IA to IC OCCC, and the survival was not affected 130. A few studies illustrated similar conclusions, reporting that patients with stage IA~IC1 OCCC were more suitable to receive FSS, and FSS would not lead to a poorer prognosis 129, 131, 132. To determine whether the indications for FSS can be extended to stage I OCCC or not, further clinical research is needed especially for patients with stage IA to IC1 OCCC strongly wishing to have babies in the future 129.

### Adjuvant Chemotherapy

Compared with other subtypes of EOC, patients with OCCC tend to be less sensitive to conventional platinum-based chemotherapy, resulting in poor outcomes. Only 11% to 27% of patients with OCCC respond to a platinum-based chemotherapy, while patients with HGSC had a significantly higher response rate of 73% to 81% 10, 92. Previous researches have indicated some mechanisms associated with this resistance, such as drugs accumulate decreasing, drugs detoxicate increasing, and DNA repair increasing. Adenosine 5'-triphosphate (ATP)-binding cassette (ABC) transporters enhance drug efflux and decrease drug accumulation, and they are important multidrug resistance factors 4. Drug activity in cell could also be reduced by drug detoxification systems. Studies have shown that the cell detoxification effect of the glutathione system is involved in the metabolism of a variety of cytotoxic drugs. Glutathione peroxidase 3 (GPx3), glutaredoxin (GLRX), and superoxide dismutase (SOD2) might result in chemotherapy resistance, which are highly expressed in OCCC 133. Two key genes, ERCC1 (excision repair cross-complementing rodent repair deficiency, complementation group 1) and XPB (xeroderma pigmentosum group B), highly expressed in OCCC, might be involved in nucleotide excision repair which related to chemotherapeutic resistance 134. DNA mismatch repair systems (MMR) are also associated with the sensitivity of DNA-damaging agents. Germline mutations of hMLH1 or hMSH2, or inactivation of somatic MMR gene usually result in loss of MMR 135. In addition, overexpression of EGFR, HNF1β, and HER2 is involved in chemoresistance and poor outcomes 4. The low proliferation activity of OCCC is also thought to be associated with chemoresistance 136.

However, platinum-based chemotherapy is still used in OCCC patients with no better choice. In addition, the effect of adjuvant chemotherapy on early-stage cancer is controversial. Several studies indicated that postoperative chemotherapy is unnecessary for patients with stage I OCCC because it does not improve survival 7, 58, 137, 138. Considering the prognosis for early-stage OCCC is good, adjuvant chemotherapy should be considered for these patients. In addition, 3~6 cycles of adjuvant chemotherapy are suggested for OCCC, though some studies have found that the duration of chemotherapy does not affect the prognosis for early-stage, non-serous tumors 120, 139, 140.

The response rate to second-line chemotherapy for recrudescent or refractory OCCC is much lower than other EOCs, even among patients who are sensitive to primary chemotherapy, with a response rate of less than 10% 10. OCCC with ARID1A-loss have been found to be more sensitive to gemcitabine than those without *in vitro*, which means OCCC with ARID1A deficiency might potentially benefit from a gemcitabine regimen for salvage therapy, with a response rate of between 22% and 60% 141, 142. It has been reported that some other regimens have demonstrated well efficacy, including paclitaxel/carboplatin, etoposide/platinum, and irinotecan/platinum, but they only increase PFS by approximately 6 months 143, 144. OCCC is an extremely chemo-resistant carcinoma, especially for patients with recurrent or refractory disease. Therefore, more effective therapies are urgent needed.

### Novel Therapeutic Strategies

#### Targeting the ARID1A gene

**Figure 3:**
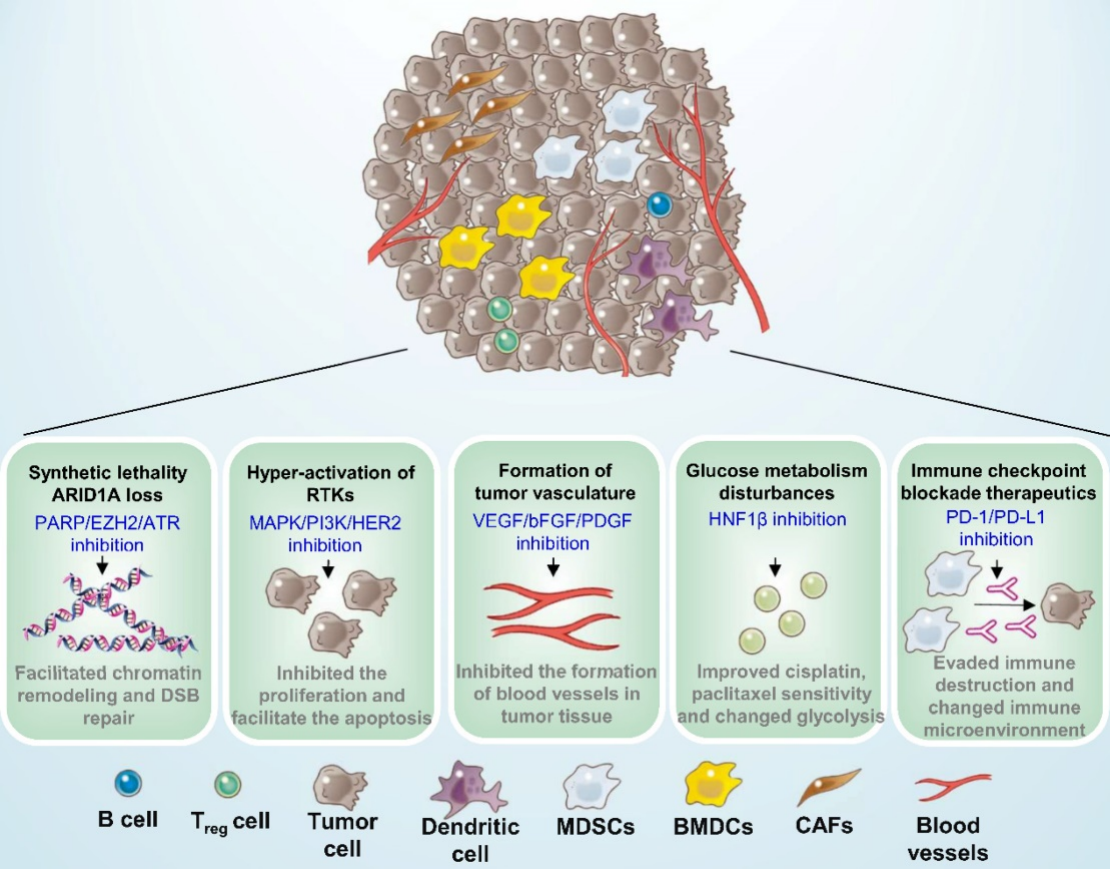
** Potential therapeutic targets for ovarian clear cell carcinoma.** Five mechanisms of potential therapeutic target have been characterized in ovarian clear cell carcinoma, including synthetic lethality with ARID1A loss, suppression of hyper-activation of RTKs, formation of tumor vasculature and glucose metabolism disturbances, and immune checkpoint blockade therapeutics. MDSCs: Myeloid-derived suppressor cells; BMDCs: Bone marrow-derived dendritic cells; CAFs: Cancer-associated fibroblasts.

ARID1A is a key component of the SWI-SNF chromatin remodeling complex and is also associated DNA double-strand break (DSB) repair. ARID1A loss occured in 40% to 57% of patients with OCCC and has been identified as an early event in the progression of malignancy, portending a poor prognosis 30. The rationale for targeting ARID1A-defective OCCC is based on synthetic-lethal approaches that can be exploited and clinically combined with targeted DNA repair proteins Poly-ADP Ribose Polymerase (PARP) or Ataxia-Telangiectasia Mutated and Rad3-related protein kinase (ATR), as well as with epigenetic factors such as enhancer of zeste homolog 2(EZH2), histone deacetylase (HDAC), or bromodomain and extra terminal (BET), shown in the **Table 1 and Figure 3.**

PARP plays an important role in single-strand DNA break repair, and inhibition of PARP plays a synthetically lethal effect, with deficient DSB repair caused by BRCA mutations 145. However, BRCA1 and 2 mutations are only observed in 2.1% of patients with OCCC, so single-agent PARP inhibitor therapy could benefit only a minority of these patients 2, 28. Because ARID1A facilitates efficient repair of DSB, Shen et al. have found that ARID1A deficiency could potentially sensitize the response of cancer cells to PARP inhibitors *in vitro* and* in vivo*, suggesting that PARP inhibitors might be a potential therapy for ARID1A-deficient OCCC through its synthetically lethal effect 146. In addition, ARID1A deficiency results in topoisomerase 2A and cell-cycle defects, increasing the dependence on ATR checkpoint activity. Therefore, ATR inhibitors are also expected to treat OCCC with of ARID1A deficiency 147. VX-970, the ATR inhibitor, has been confirmed to more than a 3-fold increased effect in multiple ARID1A-deficient cancer lines, including several OCCC cell lines 147. However, there is still no clinical trial assesses the effects of PARP or ATR inhibitors in women with OCCC, it deserves further exploration 148.

Disruption of epigenetic chromatin remodeling has an effect on driving oncogenesis of tumors that lack genomic instability. EZH2, an enzymatic component of the Polycomb Repressive Complex 2 (PRC2), regulates transcription and involves in synthetic lethal effect with ARID1A, confirmed in ovarian cancer cells and mouse models 149, 150. However, first-generation EZH2 inhibitors (DZNep) have demonstrated toxicity *in vivo*, and 2 new EZH2 inhibitors are undergoing clinical trials 151. HDAC2 interacts with EZH2 depending on ARID1A status and acts as a co-inhibitor of EZH2 to inhibit the tumor suppressor genes expression such as PIK3IP1. ARID1A-deficiency improved the sensitivity to inhibitors of pan-HDAC, for example SAHA, reducing the growth and ascites of the OCCC with ARID1A deficiency in both vivo and vitro, significant improvement of survival of mice 152. It has been reported that vorinostat, which inhibits HDAC2, a binding partner of the PRC2 complex, reduced proliferation and facilitated apoptosis in ARID1A-deficient OCCC cells 152. Inhibition of HDAC6 was also effective in several OCCC cell lines with ARID1A deficiency 153, 154. Recently, knockdown of the BET has been found to establish specific lethality in OCCC cell lines with ARID1A deficiency, inhibitors such as JQ1 and iBET762 are under clinical investigation 155. Dasatinib, a multikinase inhibitor, increased more than 2-fold sensitivity in OCCC cells with ARID1A deficiency compared with wild-type cells 156, and its therapeutic efficacy has also been confirmed in a xenograft model 157. Dasatinib is considered to be the synthetic lethality partner of OCCC with ARID1A deficiency, and the trial (GOG 283) investigating its efficacy on persistent or recurrent OCCC is still ongoing 156.

Recently, alteration of cellular metabolism in ARID1A deficient cells was put forward as a novel therapy. SLC7A11, encoding a subunit of cystine/glutamate transporter XCT, is poorly expressed in OCCC cells with ARID1A deficiency, leading to the low level of GSH and high level of reactive oxygen species (ROS). Cystine enters the cell through XCT complex to exchange glutamate and soon reduced to cysteine by thioredoxin reductase (TRXN) producing thioredoxin (TXN). Then cysteine and glutamate will be used to produce reduced glutathione (GSH) using γ-glutamate cysteine ligase (GCL). Both GSH and TXN have functions of controlling ROS levels and preventing cell death. The balance between GSH and ROS is broken in ARID1A-deficient OCCC cells, and these cells are sensitive to the inhibitors of TRXN and GSH, such as auranofin, APR-246, or buthionine sulphoximine (BSO), leading to the accumulation of ROS and decrease the antioxidant capacity, triggering cell death 158, 159. However, further research about GSH inhibitors should be developed *in vivo* and vitro, as well as combined with other target therapies to achieve better curative efficacy.

#### Downstream Pathways of Receptor Tyrosine Kinases

Receptor tyrosine kinases (RTKs) are receptors located on the surface of cells and have an important effect on proliferation, migration, differentiation, metabolism, and survival. Both the PI3K/AKT/mTOR (phosphoinositide 3-kinase/AKT/mammalian target of rapamycin) and EGF/RAS/MAPK (epidermal growth factor/RAS/mitogen-activated protein kinase) are downstream RTK pathways 160. PI3K/AKT and RTK/RAS signalling pathways activation have been proved to be involved in higher survival rate for OCCC patients 161, 162. Some genetic mutations and key components are associated with these pathways that could be potential therapeutic targets in OCCC are listed in **Table 1 and Figure 3**, including PIK3CA, PTEN, and the human epidermal growth factor receptor 2 (HER2) and MET (also known as hepatocyte growth factor receptor, HGFR).

The PI3K/AKT/mTOR pathway plays a central role to regulate cell proliferation, adhesion, apoptosis, G1 cell cycle progression, carcinogenesis, and metastasis, and activation of this pathway has an important effect on the pathogenesis of OCCC 159, 160, 163, 164. Inhibitions of this pathway have been suggested to be a promising therapeutic approach 29. The PI3K inhibitor CH51327 and the AKT inhibitor MK2206 are indicated to decrease growth of ovarian cancer cells 165, 166. Perifosine, a kind of AKT inhibitor, showed significant anti-tumor activity in OCCC that had acquired resistance to bevacizumab or cisplatin, inhibit proliferation, and induce apoptosis of OCCC cells, suggesting that AKT is a promising therapeutic target especially for recurrent OCCC after platinum-based or bevacizumab chemotherapy 167. Also, compared with HGSC, mTOR is activated in OCCC more frequently (86.6% vs 50%) 163, inhibiting mTOR can overcome OCCC resistance to cisplatin or trabectedin *in vitro* 51.

RAD001 (everolimus), an mTOR inhibitor, is a promising agent for the treatment of OCCC both as a front-line treatment and as a salvage treatment for recurrence after platinum-based chemotherapy, because the anti-tumor effect of RAD001 was greater in cisplatin resistant cell-derived tumors than in cisplatin sensitive cell-derived tumors 164. However, RAD001 would induce mTORC2-mediated AKT activation and gain resistance in RAD001-sensitive OCCC cells. Furthermore, inhibition of mTORC2 during RAD001 treatment could prevent OCCC cells from acquiring resistance to RAD001 and enhance the anti-tumor effect. Thus mTORC2-targeted therapy may be efficacious in a front-line setting as well as for second-line treatment of recurrent disease developing after RAD001 168. Recently, it has been reported that overexpression of miR-100 can inhibit mTOR signaling and enhance sensitivity to the RAD001; miR-22 is also a candidate tumor suppressor in OCCC that influence PI3K/AKT/mTOR signaling through direct and indirect effects at multiple points in the pathway^169^. However, a clinical trial of the mTOR inhibitor temsirolimus has shown unsatisfactory effects (**Table 3**) 170. ARID1A-deficient OCCC often has PI3K/AKT signaling pathway mutations, including gaining-function mutations of the PIK3CA oncogene or losing-function mutations of PTEN 5, 142, 171, and both mutations are considered early events in OCCC carcinogenesis 31. Therefore, PI3K pathway activation and ARID1A deficiency may be synthetically lethal, but requiring further investigation 148.

For the RAS-RAF-MAPK pathway, most mutations influencing this pathway occurred in the first signaling element, KRAS 29. Mutations in oncogenic KRAS often coexist with PPP2R1A (encoding serine/threonine protein phosphatase 2 scaffold subunit alpha), and thay have been observed in 9% to 20% and 7% to 15% of cases of OCCC, respectively, activating the MAPK signaling pathway 30, 142. This suggests that potential therapeutic efficacy of inhibiting the RAS signal transduction downstream in OCCC could be achieved by using MAPK and PI3K/AKT/mTOR inhibitors.

HER2, an RTK playing an important role in the regulation of proliferation, immigration and differentiation of OCCC, is overexpressed in 14% to 42.9% of cases of OCCC 31. Trastuzumab has been reported to significantly and dose-dependently inhibit tumor growth in HER2-overexpressing OCCC cell lines in mice, prolonging their survival 163, 172. However, a clinical study of the efficacy of single-agent trastuzumab found a disappointing response rate of 7.3%, with only 3 cases of complete or partial response (PR) out of the 7 cases of recurrent OCCC with HER2-overexpression 173. Therefore, combination chemotherapy with trastuzumab, which has obviously benefited patients with breast and gastric cancers, should be further investigated in patients with HER-2 overexpressed OCCC 51. MET is another RTK that participates in both MAPK and PI3K pathways and is involved in promoting proliferation and invasion in various tumors 31. MET has been found to be overexpressed in 14% to 37% of cases of OCCC, associating with a poor prognosis 51, 174. MET inhibitors have been reported to significantly inhibit the proliferation and facilitate the apoptosis of OCCC cell lines and to suppress tumor growth in xenograft models 175. However, a study of the MET inhibitor cabozantinib in 13 cases of recurrent OCCC (**Table 3**) did not demonstrate any clinical benefits (response rate = 0%) 176.

#### Targeting Angiogenesis

Anti-angiogenic drugs inhibit blood vessels formation by inhibiting vascular endothelial growth factor (VEGF), basic fibroblast growth factor (bFGF), and platelet-derived growth factor (PDGF) 29. VEGF is usually expressed in approximately 90% of cases of OCCC both in early and advanced stage, significantly higher in cisplatin-refractory OCCC cells, and related to poor prognosis. Inhibitors of VEGF or VEGF receptors (VEGF-R) have been the typical therapy for EOC 142, 177, 178, with a high response rate combined with chemotherapy regimen of carboplatin and paclitaxel (**Figure 3**) 179. To date, clinical trials of bevacizumab (targeting VEGF), sunitinib (targeting VEGF-R and PDGF-R), and carbonatitic (targeting VEGF-R and MET) have been completed but have shown unsatisfactory effects (**Table 3**). However, receiving ENMD-2076, a multi-target kinase inhibitor of Aurora kinase-A, VEGFR, and FGFR, patients with ARID1A-deficient OCCC had a significantly higher 6-month rate of PFS (33% vs 12%), compared with the ARID1A-positive cases in a phase II clinical trial 180. Nintedanib is a new, effective, triple-angiokinesis inhibitor which is mainly targeted at VEGFR 1-3, FGFR 1-3, and PDGF receptors α and β. A clinical trial of NiCCC (ENGOT-GYN1) is also ongoing 181. In a recent preclinical research, axitinib, an inhibition of VEGFR signals, showed significant anti-tumor effects in OCCC cells associated with cell proliferation, apoptosis and migration* in vivo* and *in vitro*. However, the effects of axitinib were not promising against drug-resistant EOC, so the clinical effects of combination therapies of axitinib with other target agents and immunotherapy are still need to be identified 182.

#### Targeting Hepatocyte Nuclear Factor‑1β

There is severe oxidative stress in the endometriotic cysts microenvironment, so the epithelial cells suffer from more cellular and DNA damage and develop spontaneous malignant transformation 183. A transcription factor, hepatocyte nuclear factor‑1β (HNF1β), is expressed in more than 95% of cases of OCCC and is regarded as a biomarker of OCCC 33. HNF1β facilitates proliferation of OCCC cells but inhibits their invasion and migration, which is a possible mechanism for the common early-stage manifestations of OCCC 51. Overexpression of HNF1β in OCCC promotes glucose uptake and glycolysis, dramatically changing cellular metabolism to enhance oxidative stress resistance and benefiting cell survival 184. Thus, glucose metabolism could be the therapeutic target of HNF1β. Overexpression of HNF-1β is also shown to reduce ROS and protect cancer cells from the internal oxidative stress caused by the drastic changes in their cellular metabolism. It is proposed that HNF-1β may be pivotal for cancer cell survival due to antistress effects, rather than increased proliferative potential 185. Furthermore, HNF1β is considered one of the factors contributing to resistance in OCCC, and knockdown of HNF1β can significantly improve cisplatin and paclitaxel sensitivity 186, 187. HNF1β inhibitors, microRNA mir-802 187, may play a therapeutic role by destroying oxidative stress resistance (**Figure 3**) 5. In addition, metformin has also been shown to depress cancer growth and promote paclitaxel sensitivity in mice 188. HNF1β is a vital potential therapeutic target for OCCC whereas effective HNF1β inhibitors have not been found, and it has been commonly used as IHC biomarker.

#### Immune Checkpoint Inhibitors

It is currently believed that the immune microenvironment plays an important role in development and pathogenesis of tumors. The ability to evade immunity makes the immune microenvironment an emerging characteristic of cancer, and immune checkpoint blockade therapy has become more popular worldwide 29. Up-regulation of several inflammatory cytokines and immune-related gene, as well as IL-6, STAT3 related genes is suggestive of an immune-suppressive microenvironment which may be associated with sensitivity to immune checkpoint inhibitors in OCCC. It has been indicated that effector memory CD8+ T cell phenotype, cytotoxic T lymphocyte-associated antigen-4 (CTLA-4), programmed death 1 (PD-1), T cell immunoglobulin and mucindomain containing-3 (Tim-3), and lymphocyte-activation gene-3 (LAG3) genes were overexpressed in OCCC, whereas expression of human leukocyte antigens (HLA) -A, -B, and -C were decreased. These changes result in an immune-suppressive microenvironment which may serve as a promising therapeutic target in OCCC 185. Several clinical trials of PD-1 and programmed death ligand 1 (PD-L1) monoclonal antibodies are outlined in **Table 3 and Figure 3** 189-193. In the 4 completed trails, avelumab, durvalumab+cediranib, pembrolizumab, and nivolumab have demonstrated significant efficacy and revealed the potential therapeutic efficacy as immune checkpoint inhibitors. Although these clinical trials had small OCCC sample sizes and needs further verification, anti-PD-1 or -PD-L1 antibody is a promising novel therapeutic drug for OCCC patients. A previous analysis found PD-L1 expression defects and mismatch repair (MMR) defects occurred in 43% and 67% of OCCC cases, respectively 194. MMR deficiency is considered to be a predictive marker for immunotherapy such as anti-PD-1 and -PD-L1 antibodies, with a high sensitivity 195, 196. Glypican-3 (GPC3), a member of the glypican family of heparan sulfate proteoglycansone, is a potentially useful carcinoembryonic antigen for cancer vaccine immunotherapy and is overexpressed in OCCC 185. In two small studies, OCCC patients were treated with a GPC3-derived peptide vaccine, and the overall response rate was reported as 9.4% (2 PR and 1 stable disease) 82 with the disease control rate of 17.9% in 32 patients 197. Microsatellite instability (MSI) high tumors are associated with an enriched tumor mutation burden and a highly immunogenic phenotype. Women with Lynch syndrome, and a germline mutation of the mismatch repair genes (i.e., MLH1, MSH2, MSH6, and PMS2), have an increased life-time risk of colorectal, endometrial and ovarian cancer, including OCCC as well as endometrial ovarian carcinoma. Thus, Lynch syndrome should also be taken into consideration for the OCCC patients, especially with a Lynch syndrome related family history 185. Therefore, the efficacy of immunotherapy in patients with OCCC should be further investigated in future clinical trials.

#### Other potential therapeutic targets

In addition to the targeted therapies described above, there are several promising therapeutic targets confirmed in clinic trials and preclinical studies. Lurbinectedin, a new agent that targets active transcription, exhibits antitumor activity in OCCC when used as a single agent and has synergistic antitumor effects when combined with irinotecan both as a first-line treatment and as a salvage treatment for recurrent lesions that develop after platinum-based or paclitaxel treatment 198. The Japanese Gynecologic Oncology Group conducted the first randomized phase III, OCCC-specific clinical trial that compared irinotecan and cisplatin (CPT-P) with paclitaxel plus carboplatin (TC) in patients with OCCC, but no significant survival benefit was found for CPT-P 199. Ferroptosis is associated with various pathological conditions, including acute kidney injury, hepatocellular degeneration and hemochromatosis, traumatic brain injury, and neurodegeneration. OCCC had an intrinsic vulnerability to glutathione peroxidase 4 (GPX4) inhibition-induced ferroptosis through the hypoxia-inducible factor (HIF) pathway, thus GPX4 was suggested as a therapeutic target in OCCC 200. However, due to the poor bioavailability of current small-molecule GPX4 inhibitors, the efficacy of chemical inhibition of GPX4 remains to be demonstrated. ADP-ribosylation factor-like protein 4C (ARL4C) is also a potential therapeutic target in OCCC, which contributed to infiltration, metastasis, and chemotherapeutic resistance in OCCC via molecular mechanisms such as epithelial-to-mesenchymal transition (EMT). Statins and bisphosphonates have a potential in ARL4C-targeted therapy in OCCC, but further studies are needed to verify its applicability 201. EGLN (Egg-laying defective nine homolog) is a direct downstream sensor of oxygen tension. In OCCC, knockout or pharmacological inhibition of EGLN1 could stabilize HIF1A and inhibit proliferation *in vitro* and *in vivo*, suggesting that EGLN1 inhibition is a potential therapeutic strategy in OCCC 202.

There are still some potential targets need to be further explored, such as Fatty acid synthase (FASN), a potential downstream target of NAC1 in OCCC, and OCCC with FASN overexpression were more sensitive to a potent FASN inhibitor 203.By inhibition of anti-apoptotic protein MCL1 and BCL-XL/BCL-2, co-treatment with PF271and ABT-737 was profoundly effective in inducing apoptosis of OCCC cell lines 204. Suppression of ANXA4, which involved in proliferation, chemoresistance, and migration and invasion of OCCC, might be a potential target therapy of OCCC 205 Deregulation of Rho GTPases was common among OCCC, participating in tumorigenesis, invasiveness and metastasis. Several inhibitors targeting effectors and activators of the Rho GTPases are available, and their potential role in OCCC remains to be explored 206 LEFTY overexpression has anti-tumor effects in cellular proliferation and apoptosis which could helpful for therapy of OCCC 101. In OCCC, miR-9 overexpression may affect pathogenesis by targeting E-cadherin, thereby inducing an epithelial-mesenchymal transition. Therefore, miR-9 may be also a promising therapeutic target strategy 207.

## Prognosis

### Primary OCCC

The prognosis of early-stage OCCC is obviously superior to advanced OCCC. The 3-year survival rates for OCCC at stage I, II, III and IV are 80%, 47%, 34%, and 30% for PFS, and 96%, 85%, 54%, and 40% for OS, respectively 6, 7. Among patients at early-stage, survival is better in OCCC compared to HGSC, but advanced-stage OCCC has worse outcomes compared with HGSC 208. Intraoperative tumor rupture could lead to an iatrogenic stage increase of tumors from IA or IB to IC1, but the overflow of tumor cells during surgery does not seem to have an adverse impact on survival outcomes in stage I OCCC. Survival outcomes for stage IC1 OCCC have been reported to be similar to that of IA and IB, and are better than IC2 and IC3 208. The high rupture rate may be due to the related pelvic endometriosis, which leads to the formation of benign adhesions, making it hard to dissect tumors 106, 208. However, in primary oophorectomy, it is still necessary to avoid tumor rupture 209.

In addition to FIGO stage, resistance to chemotherapy is also an independent prognostic factor in OCCC. For platinum-based chemotherapy, the overall response rate in OCCC is significantly lower than other types of EOC. Thus, compared with patients receiving non-platinum chemotherapy, platinum-based chemotherapy does not seem to improve the survival of OCCC patients 10, 92.

The extent of cytoreduction is another major factor in determining the prognosis of OCCC patients 210. Optimal debulking is regarded to be very important, and significantly poorer prognosis has been observed, even in patients with small-volume disease 120. Recurrent OCCC has much poorer survival 211.

Retroperitoneal lymph node metastasis is an effective indicator for decreased OS 72, and the lymph node ratio (LNR), which is the ratio of the number of metastatic lymph nodes (MLNs) to the number of resected lymph nodes (RLNs), is considered an independent prognostic predictor. Nie et al. verified that patients with advanced OCCC and an elevated LNR (>25%) have unfavorable prognosis 212.

Patients with pathologically confirmed endometriosis-associated OCCC are 4 to 8 years younger at diagnosis than those without endometriosis 53, 213, 214, and they are more likely to diagnosed at early-stage manifestations and have higher probability of optimal debulking, lower CA-125 levels, and a higher rate of platinum-sensitivity 19, 45, 46, 48. Patients with endometriosis-associated OCCC also tend to have lower percentages of LNM (15% to 17% vs 40% to 41%) and lymphovascular space invasion (LVSI) (23% vs 51%) compared with those without endometriosis-associated OCCC 46, 72. The rate of 5-year OS in patients with endometriosis is 70.2% to 74.1%, compared with 46.4% to 52.6% for those without endometriosis, but most studies have concluded that the presence of endometriosis is not an independent prognosis factor after adjusting related clinical factors 46, 53, 72, 215.

VTE also adversely impacts patient survival, even after adjusting FIGO stage, with a shorter median OS (19 to 54 vs 90 to 140 months) and PFS (11 to 17 vs 43 to 76 months) compared with the cases without VTE, and its risk of recurrence and death increased by 3.9 ~ 6.3 times 77, 90. The occurrence of VTE during primary therapy is also an independent predictor for prognosis 90. Thus, it has been recommended that patients with OCCC may benefit from long-term anticoagulation. In addition, older age, positive peritoneal cytology, ascites, and omental involvement have also been found to be independent prognostic factors of OCCC 72, 216.

In addition, there are some other promising prognosis predictors. Recently, ten novel histotype-specific prognostic biomarkers for OCCC were reported, positive ARPC2, GNB1, KCTD10, NUP155, RPL13A, SETD3, SMYD2, and TRIO were related to poorer outcomes, whereas positive CCT5 and RPL37 were related to better prognosis 217. In addition, high gene expression for CCNB2, CORO2A, CSNK1G1, FRMD8, LIN54, LINC00664, PDK1, and PEX6, were associated with shorter PFS for OCCC and endometrial ovarian carcinoma patients 218. The high frequencies of neoantigens per somatic mutation (neoAg frequencies), rather than overall mutational load or number of predicted neoantigens per se, is an independent prognostic factor for decreased clinical outcome and low expression of immunity-associated genes 219. Up-regulated rs4873815-TT/ZNF707, ARL4C, mitochondrial superoxide dismutase (SOD2) expression are predictive biomarkers for worse prognosis in OCCC 201, 220, 221. And NAC1/FASN expression is a biomarker of poor outcome for patients treated with conventional platinum-based chemotherapy in OCCC by modulates sensitivity of ovarian cancer cells to cisplatin 203.

### Recurrent OCCC

Recurrence rates have been reported to be 29%, 30%, 62%, and 73% for patients with OCCC at stages I, II, III and IV, respectively 15, 104. Patients with recrudescent OCCC have an extremely poor prognosis, with a 5-year survival rate of merely 13.2% and a post-recurrence survival of 10.0 months, significantly lower than those with recurrent serous carcinoma (18.2% and 18.9 months) 104, 105. This is caused by the lower chemotherapy response rate and the absence of valid therapy for recurrent disease. After first-line chemotherapy, only 1% to 10% relapsed platinum-resistant OCCC patients could response to various second-line chemotherapy regimens 10, 33. Despite adopting novel chemotherapy regimen and the investigation of novel combinations, there has been little improvement during the past few decades 105. In Crotzer's research, of the 22 OCCC patients with platinum-sensitivity, 2 patients (9%) showed partial response to treatment with paclitaxel plus carboplatin, while in 83 OCCC patients with platinum-resistance, only 1 patient (1%) showed partial response 222. On the contrary, Takano et al. showed that even in the platinum-sensitive setting, response to chemotherapy was observed in only 8% (2 of 24) of patients 211. Currently, there is no evidence to support whether chemotherapy plus cytoreduction is better than salvage chemotherapy alone for recurrent OCCC 105.

### Surveillance of High-Risk Groups

In terms of surveillance, patients with a higher risk of OCCC should be closely monitored, including those with a long-term endometriosis, early menarche, late menopause, and a history of infertility associated with endometriosis, infertility treatment and ovarian endometrioma 223. Owing to the poor prognosis of OCCC, asymptomatic patients should also pay attention to the regular gynecologic surveillance. Surgery should be considered if the tumor grows in regular follow-up 6, 10, 11. The median time between diagnosis of endometriosis and diagnosis of OCCC is 50 months (ranging from 12 to 213 months). In consideration of the timing of cancer diagnosis and the duration of malignant transformation, Son et al. recommended that active surveillance to be considered beginning at age 35 years in patients with endometriosis, with examinations conducted at least once a year 215.

## Conclusion

OCCC is a special pathological subtype of EOC that has its origin in endometriosis. The prognosis for advanced-stage OCCC is extremely poor because it has inherent platinum-resistance, so it is urgent to investigate more effective therapies. In this review, we summarized the pathogenesis, clinical features, molecular classification, diagnostic methods, future potential targeted treatments and prognosis biomarkers for OCCC. On the basis of the unique molecular characteristics of OCCC, several molecular-targeting drugs and immunotherapies have been extensively investigated in combination with conventional chemotherapy regimens. Although many combination therapy trials are currently underway or have been completed, precision therapy such as the PARP inhibitors and PD-1/PD-L1 antibodies may play an important role in OCCC treatment because it has been shown to be effective in HGSC and other cancers. However, personalized therapy based on the differences in genetic and molecular characteristics should also be pursued. Recent advances in molecular analysis of the clinical features of OCCC might contribute to future advances in the diagnosis and treatment of OCCC.

## Figures and Tables

**Table 1:** Genetic alterations and possible molecular targets in ovarian clear cell carcinoma

Gene	Type of alterations	Frequency (%)	Original function	Therapeutic selection	Drug	Reference
ARID1A	Mutation copy number loss	40-57%	A key component of the SWI-SNF chromatin remodeling complex; involved in DNA double-strand break (DSB) repair.	Inhibition of: PARP, EZH2, ATR, HDAC2, HDAC6, BET, BCR/ABL/SRC, ROS induction.	Talazoparib, Olaparib, GSK126, DZNep, AZD6738, VX-970, Vorinostat, ACY1215, JQ1, iBET762, Dasatinib, Elesclomol	29, 31
PIK3CA	Mutation	20-51%	Activating the PI3K	PI3K, mTOR signal inhibitor	Buparlisib, copanlisib, Temsirolimus	30, 160, 171
PTEN	Loss-of-function mutation	5-8.3%	A tumor suppressor that negatively regulates the PI3K-AKT-mTOR pathway.	mTOR signal inhibitor.	Temsirolimus	5,142
KRAS	Mutation	9-20%	Activation of RAS/RAF/MEK/ERK, PI3K-AKT-mTOR and RAS/RAF/MAPK pathways.	MAPK signal inhibitor.	-	29
PPP2R1A	Mutation	7-15%	Impaired PP2A function leading to uncontrolled cell growth.	MAPK signal inhibitor.	-	30, 142
HNF1β	Hypo-methylation overexpression	>90%	Facilitating glucose uptake and glycolysis to change cellular metabolism.	Glucose metabolism.	Mir-802, metformin	33, 51, 184
BRCA1/2	Mutation	6.7%	DSB repairing deficiency.	PARP inhibitor.	Olaparib	2, 28

**Table 2:** Comparison of ovarian clear cell and endometrioid carcinoma

		Ovarian clear cell carcinoma	Ovarian endometrioid carcinoma
	Prevalence	5%-10% of epithelial ovarian carcinoma^†^	
Similarities	General classification	Epithelial ovarian carcinoma	
	Molecular subtyping	Type I	
	Origin	Endometriosis	
	Clinical characteristics	Diagnosed younger and at an earlier stage	
	Prognosis	Better at early stage, poor at advanced stage or recurrence	
	Platinum sensitivity	Low	
Differences	Pathological grade	Not recommended to grade	Low, intermediate, and high
	Molecular characteristics	ARID1A and PIK3CA gene mutation, HNF1β overexpression	PTEN, KRAS, p53, and β-catenin gene mutations
	Original types of endometriosis cells	Ciliated cell lineage	Secretory cell lineage
	ER and PR	Low expression	High expression

†The prevalence of ovarian clear cell carcinoma is 5-10% of epithelial ovarian carcinoma in America, but with a higher percentage in East Asia. Abbreviation: ER, estrogen receptor; PR, progesterone receptor.

**Table 3:** Clinical trials of target therapy for patients with ovarian clear cell carcinoma

Therapeutic targets	Drug	Trial name	Patients type	No. of OCCC	Response summary	Reference
Synthetic lethality ARID1A loss	Dasatinib	GOG 283	Persistent or recurrent OCCC	-	On-going (NCT02059265)	156
mTOR	Temsirolimus +paclitaxel +carboplatin	GOG 268	Stage III to IV OCCC	90	12-months PFS rate=54%, not significantly different than historical controls; Most common grade 3-4 adverse events were cytopenia; Otherwise well tolerated.	170
VEGFR, PDGF	Sunitinib	GOG 254	Persistent or recurrent OCCC	30	Response rate of 6.7%, Median PFS=2.7 months, OS=12.8 months Grade 4-5 adverse events: thrombocytopenia (5), anemia (2), acute kidney injury (1), stroke (1), and allergic reaction (1).	177
VEGFR, PDGFR, FGFR	Nintedanib	NiCCC (ENGOT-GYN1)	Persistent or recurrent OCCC	-	On-going (NCT02866370)	181
MET, RET, VEGFR2	Cabozantinib	NRG-GY001	Persistent or recurrent OCCC	13	Response rate of 0%; Median PFS=3.6 months, OS=8.1 months; Single patient with lethal thromboembolic event, possibly treatment related.	176
Aurora A, VEGFR, FGFR	ENMD-2076	A Study of ENMD-2076 in OCCC	Persistent or recurrent OCCC	40	Response rate of 7.5% higher 6-month PFS rate in ARID1A protein loss than ARID1A IHC positive population (33% vs. 12%); Adverse events were well tolerated.	180
VEGF	Bevacizumab +Carboplatin +Paclitaxel	JGOG 3022	Stage III to IV epithelial ovarian/fallopian tube/primary peritoneal cancer.	11	Response rate of 63.6%; Median PFS=12.3 months, 1-year PFS=50.5 months; Adverse events were well tolerated.	179
PD-L1	Avelumab	JAVELIN Solid Tumor Trial	Recurrent or refractory stage III-IV ovarian cancer.	2	1 had a partial response and the other had an immune-related partial response.	189
PD-L1, PARP or VEGFR	Durvalumab+ Olaparib or Cediranib	NCT02484404	Recurrent or metastatic measurable solid malignancies.	1	This patient received durvalumab + cediranib exhibited a partial response.	190
PD-1	Pembrolizumab	KEYNOTE-100	Recurrent ovarian cancer.	19	Response rate of 15.8%; Adverse events were well tolerated.	191
PD-1	Nivolumab	NCT02484404	Advanced or relapsed, platinum-resistant ovarian cancer.	2	1 case of complete response.	192
PD-1/PD-L1	Durvalumab	MEDI-4736	Persistent or recurrent OCCC.	46	On-going (NCT03405454).	193

Abbreviation: OCCC, ovarian clear cell carcinoma; PFS, progression-free survival; OS, overall survival; IHC, immunohistochemistry.
